# Analysis of non-motor symptoms in amyotrophic lateral sclerosis

**DOI:** 10.1080/21678421.2023.2280618

**Published:** 2023-11-19

**Authors:** Ali Shojaie, Ahmad Al Khleifat, Payam Sarraf, Ammar Al-Chalabi

**Affiliations:** 1 Maurice Wohl Clinical Neuroscience Institute, Institute of Psychiatry, Psychology and Neuroscience, King’s College London, London, UK; 2 Department of Neuromuscular Diseases, Iranian Centre of Neurological Research, Neuroscience Institute, Tehran University of Medical Sciences, Tehran, Iran, and; 3 Department of Neurology, King’s College Hospital, London, UK

**Keywords:** Amyotrophic lateral sclerosis, non-motor symptoms, questionnaire, pain, cold limbs

## Abstract

**Objective:**

We investigated non-motor symptoms in ALS using sequential questionnaires; here we report the findings of the second questionnaire.

**Methods:**

A social media platform (Twitter, now known as X) was used to publicize the questionnaires. Data were downloaded from SurveyMonkey and analyzed by descriptive statistics, comparison of means, and regression models.

**Results:**

There were 182 people with ALS and 57 controls. The most important non-motor symptoms were cold limbs (60.4% cases, 14% controls, *p* = 9.67 x 10^−10^) and appetite loss (29.7% cases, 5.3% controls, *p* = 1.6 x 10^−4^). The weaker limb was most likely to feel cold (*p* = 9.67 x 10^−10^), and symptoms were more apparent in the evening and night. Appetite loss was reported as due to feeling full and the time taken to eat. People with ALS experienced medium-intensity pain, more usually shock-like pain than burning or cold-like pain, although the most prevalent type of pain was non-differentiated.

**Conclusions:**

Non-motor symptoms are an important feature of ALS. Further investigation is needed to understand their physiological basis and whether they represent phenotypic differences useful for subtyping ALS.

## Introduction

Amyotrophic lateral sclerosis (ALS), also known as motor neuron disease, is a progressive neurodegenerative disease primarily characterized by the loss of motor neurons in the brain and spinal cord, resulting in muscle weakness and atrophy. However, ALS also manifests with a range of non-motor symptoms that significantly impact quality of life. These non-motor symptoms further complicate the management of ALS, highlighting the importance of a comprehensive approach that addresses the disease’s motor and non-motor aspects to provide optimal care and support to individuals affected by ALS (1, 2).

Patients diagnosed with ALS experience a wide range of symptoms including pain and autonomic, gastric, vascular, and psychiatric symptoms. People with ALS are also prone to sleep disturbance (3–5). These symptoms are referred to as non-motor symptoms. However, the definition is not straightforward, as some non-motor symptoms are a direct result of motor weakness, while others are likely a result of neurodegeneration outside the motor system, and while this distinction is important from a pathological point of view, it may not be for someone experiencing such symptoms.

The situation is complicated further, since motor symptoms in ALS, such as muscle weakness and atrophy, can significantly impact non-motor symptoms regardless of their basis. The loss of motor neurons affects not only voluntary movements of the limbs and trunk, but also respiratory difficulties, swallowing problems, and speech impairment (6). These physical limitations can contribute to psychological and emotional distress, including anxiety, depression, and social isolation. Furthermore, the progressive nature of ALS and the increasing reliance on caregivers for activities of daily living can further exacerbate non-motor symptoms, highlighting the intricate interplay between motor and non-motor aspects of the disease. For clinicians, in addition to having a substantial influence on the quality of life, non-motor symptoms may also serve as endophenotypes and have pre-diagnostic significance as observed in other neurodegenerative conditions like Parkinson’s disease (7).

Due to the prominence of motor symptoms in ALS, additional symptoms are frequently underreported by patients or may be disregarded by medical professionals. For example, the relationship between frontotemporal impairment or dementia and ALS was unnoticed for many years, until the evidence was made overwhelming by genetic analyses even though it was well-recognized in some circles before that (8) and carers would report personality changes to doctors (9).

A quantitative comparison of studies focused on different aspects of ALS based on publication counts reveals that non-motor symptoms account for only 0.4% of such papers (5). A search of the PubMed database using the keywords “amyotrophic lateral sclerosis” and “non-motor symptoms” generated 131 papers dedicated to the study of non-motor symptoms in ALS, compared with a total of 34,346 publications on ALS as a whole on 25/08/2023.

The impact of non-motor symptoms on persons with ALS is comparable to, and in some cases higher, than that of motor symptoms, because of the impact on quality of life. We have previously examined the relative frequencies of non-motor symptoms in people with ALS and controls (10). Here we report the outcomes of questions for non-motor symptoms that we previously found to be more frequent than expected in people with ALS.

## Materials and methods

An initial 20-item questionnaire was developed covering the domains of autonomic function, sleep, pain, gastrointestinal disturbance, and emotional lability (5). This study is based on a second questionnaire following the results of the initial questionnaire to focus more on the symptoms patients suffer the most. This second questionnaire was posted through the online system, SurveyMonkey. A social media platform (Twitter) was used to publicize a link to the questionnaire. To target people with amyotrophic lateral sclerosis (ALS), tweets were made from accounts with high numbers of followers with MND/ALS and with hashtags to increase visibility to those searching for ALS, but the invitation explicitly also stated that all were welcome to participate. The first questionnaire was made available for a total of 20 weeks from February 2022 to July 2022 and the second, used in this study, from November 2022 to January 2023.

For the domains identified in the first questionnaire, questions for a second questionnaire were based on responses from the first questionnaire and the range and nature of symptoms reported in the clinic. For pain, additional questions were taken directly from the UK Biobank pain and sensation scale https://biobank.ndph.ox.ac.uk/showcase/ukb/docs/pain_questionnaire.pdf.

In order to test if our data were consistent with expectations for ALS, we examined the male/female ratio and age of onset in cases.

### Statistical methods

We compared the frequency, location, severity, and nature of the symptoms (such as the type of pain) between patients and the control group. Chi-square statistics and *t-*tests were used to compare reported symptoms in cases and controls. Frequencies of responses as well as absolute counts are reported. Because we explored subgroups of symptoms, absolute numbers are small and most comparisons in subgroups are therefore descriptive and shown in tables. All analyses were performed in SPSS v25.0 (SPSS Inc, Illinois, USA)

## Results

There were 182 cases and 57 controls. A further 12 respondents did not provide that information and had incomplete questionnaires. They were removed from further analysis.

As expected, there were proportionately more males in the ALS group than the control group, with 56% males in the cases and 30% in the controls (chi-square = 11.3 1df, *p* = 7.86 × 10^−4^). Similarly, there was a difference in ages, with cases at onset being a little older than controls at the time of answering the questionnaire, with mean age in cases: 50.84 (95% CI 50.77, 53.88) and mean age in controls 46.49 (95% CI 46.34, 46.73, *p* = 3.13 × 10^−5^).

Cold limbs and loss of appetite showed the biggest differences between cases and controls (Table 1). Urinary urgency was very common in both groups and more common than expected in the controls, suggesting that there may have been difficulty in respondents interpreting the question as asked. The results were otherwise similar to those we found in the responses to Questionnaire 1.

**Table 1.** Distribution of symptoms among cases and controls in the study population.

	Cold limbs	Pain	Sleep problems	Loss of appetite	Urge to go toilet
Cases % responding “yes”	60.4	57.7	43.4	29.7	93.1
Cases n responding “yes”	110	105	79	54	174
Cases n responding “no”	72	77	103	128	8
Controls % responding “yes”	14.0	33.3	38.6	5.3	80.7
Controls n responding “yes”	8	19	22	3	46
Controls n responding “no”	49	38	35	54	11
Chi square statistic and p-value	37.39, 9.67 × 10^-10^	10.32, 0.0013	0.412, 0.52	14.24, 0.00016	13.1, 0.00030

The frequency and time patterns of limb coldness and pain were investigated. In patients experiencing a cold limb, coldness is experienced by 64.5% at a specific time of day, which is similar to the proportion in controls although counts are too small for formal comparison (Table 2). In patients, coldness primarily occurred in the weaker limb as opposed to the strong limb and tended to occur more in the evening and night (Tables 2 and 3).

**Table 2.** Cold limbs in cases and controls, as well as the timing pattern of coldness.

Cold limbs	Coldness experienced at any time without a pattern	Coldness experienced with a specific daily pattern	Morning	Afternoon	Evening	Night
Cases %	64.5	35.5	–	–	–	–
Cases n	71	39	6	8	12	13
Controls %	62.5	37.5	–	–	–	–
Controls n	5	3	0	1	1	1

**Table 3.** The distribution of cold limbs in patients and controls depending on limb strength.

Cold limbs	Feeling cold in weak limb	Feeling cold in both weak and strong limbs	Feeling cold in strong limb	Do not have weak limbs
Cases %	61.8	30	2.7	5.5
Cases n	68	33	3	6
Controls %	3.5	1.7	8.8	86.0
Controls n	2	1	5	49

Table 4 shows various types of pain. In patients, shock-like pain was more common than burning or cold-like pain, but the most frequent was an undifferentiated form of pain. Most patients reported medium intensity pain whereas, for controls, the distribution among the small numbers responding was relatively even (Table 5). Some pain was accompanied by additional symptoms including numbness, itching, burning, and pins and needles, which reveals that patients are more likely to experience pain that was not included in the questionnaire (Table 6).

**Table 4.** The various types of pain reported. Respondents could report multiple pain types and the total percentage does not sum to 100.

Pain	Burning	Cold	Shock	Some other type
Cases %	33.3	17.1	24.8	46.7
Cases n	35	18	26	49
Controls %	21.1	94.7	21.1	68.4
Controls n	4	18	4	13

**Table 5.** Pain intensity in patients and controls on a scale of 1 to 10, with 10 being the most severe pain. The scale only refers to the types of pain reported in Table 4.

Pain scale	1	2	3	4	5	6	7	8	9	10	No response
Cases %	2.9	5.7	12.4	22.8	11.4	19.0	7.6	4.7	7.6	2.9	2.9
Cases n	3	6	13	24	12	20	8	5	8	3	3
Controls %	0	15.7	10.5	15.7	5.2	10.5	5.2	5.2	5.2	5.2	21.0
Controls n	0	3	2	3	1	2	1	1	1	1	4

**Table 6.** Sensations felt in addition to pain. Respondents could report multiple associations and the total percentage may not sum to 100.

	Pain with a burning sensation	Pain with pins and needle sensation	Pain with numbness	Pain with itching	Other kind of pain
Cases %	17.5	21.81	20.60	11.51	28.48
Cases n	29	36	34	19	47
Controls %	20.0	16.0	24.0	0	40
Controls n	5	4	6	0	10

Cases have higher appetite loss than controls (29.7% cases and 5.3% controls). Feeling full quickly and the time taken to eat were the main reasons for losing appetite in patients (Table 7). Numbers with loss of appetite in controls were too small for a meaningful analysis.

**Table 7.** Different reasons for loss of appetite. Respondents could provide more than one reason for loss of appetite and the total percentage does not sum to 100%.

Loss of appetite	Feeling full quickly	Low mood	Nausea	Time it takes to eat	Medication	Other reasons
Cases %	25.5	45.7	8.5	23.4	6.3	15.9
Cases n	24	19	8	22	6	15
Controls %	22.2	22.2	11.1	11.1	11.1	22.2
Controls n	2	2	1	1	1	2

We also looked into sleep issues and the urge to use the toilet. There was no difference between groups in reporting sleep issues, and urinary urgency was reported as very frequent in both groups. We did not therefore further analyze these responses.

## Discussion

We have shown that non-motor symptoms are frequent in people with ALS, with the most commonly reported being loss of appetite, limb pain, and limb coldness. Although these are also reported by the control group, all are particularly frequent in ALS. Although urinary urgency was also reported as a frequent symptom, it was also very frequent in controls, and we did not therefore analyze this further, as the pattern of responses suggests a misinterpretation of the question. Both groups reported a similar frequency of sleep disturbance.

Most often, patients reported cold limbs in the weaker limb at any time of day without any discernible pattern. The emphasis on the weaker limb is consistent with immobility being the cause, but also with the possibility of autonomic neuropathy alongside motor neuron loss, demonstrating the need for further research. If immobility is a contributor, then physiotherapy, passive movements, and massage could help, as well as the use of a blanket. If autonomic dysfunction is responsible, employing neuropathic medications like gabapentin might be appropriate (11,12).

Although patients feel pain similar to electrical shock, cold pain, and burning pain more frequently than other types of pain, most do not fall into one of these groups and instead experience some other pattern. Of the 58% of people with ALS reporting pain, most reported moderate pain rather than severe or mild, with 65% scoring pain levels at 4, 5, 6, or 7 on a 10-point scale. Only people with ALS reported pain with itching, with 19 doing so. Other associated sensations were not obviously different between cases and controls, but the numbers are small and therefore firm conclusions cannot be drawn. Itching has been previously reported as an important pain-associated symptom in ALS (13) and strategies for treating pain in ALS developed (https://www.mndassociation.org/sites/default/files/2022-11/P11%20Pain.pdf).

The two primary causes of loss of appetite in patients are low mood and taking a long time to eat (Table 7). Perhaps surprisingly, medication use is not a strong cause and is not obviously different between patients and controls. Strategies for addressing loss of appetite should therefore consider addressing mood, and focus on assisting people to eat and allowing sufficient time to eat. This could take the form of excellent nursing care in assisting patients to consume adequate food and fluid intake, and taking medications to increase appetite may not be essential (14). Another possible strategy could include having less food to consume each meal, but more servings throughout the day.

This study has several important limitations. The results are based on a questionnaire, and the diagnosis, nature of responses, and accuracy of responses cannot be verified. The small number of responses from people without ALS means that statistical comparison is not possible for subgroup analyses, and furthermore, strongly suggests bias, since respondents are more likely to have symptoms to report, or may have personal links to people with ALS. Nevertheless, this is an exploratory step in the detail of non-motor symptoms experienced by people with ALS and therefore has value if the limitations are borne in mind.

Non-motor symptoms significantly negatively influence patients’ quality of life and are therefore important to address and manage in the clinic. As we learn more, they may become useful as early diagnostic criteria, as endophenotypes, or to help classify ALS.

## Data Availability

Not applicable.
